# Unimolecular Decomposition Reactions of Picric Acid and Its Methylated Derivatives—A DFT Study

**DOI:** 10.1021/acs.jpca.1c10770

**Published:** 2022-04-26

**Authors:** Kristine Wiik, Ida-Marie Høyvik, Erik Unneberg, Tomas Lunde Jensen, Ole Swang

**Affiliations:** † Chemistry Department, The Norwegian University of Science and Technology (NTNU), Høgskoleringen 5, 7491 Trondheim, Norway; ‡ Norwegian Defence Research Establishment (FFI), P.O. Box 25, 2027 Kjeller, Norway; § Department of Process Technology, SINTEF Industry, P.O. Box 124 Blindern, 0314 Oslo, Norway

## Abstract

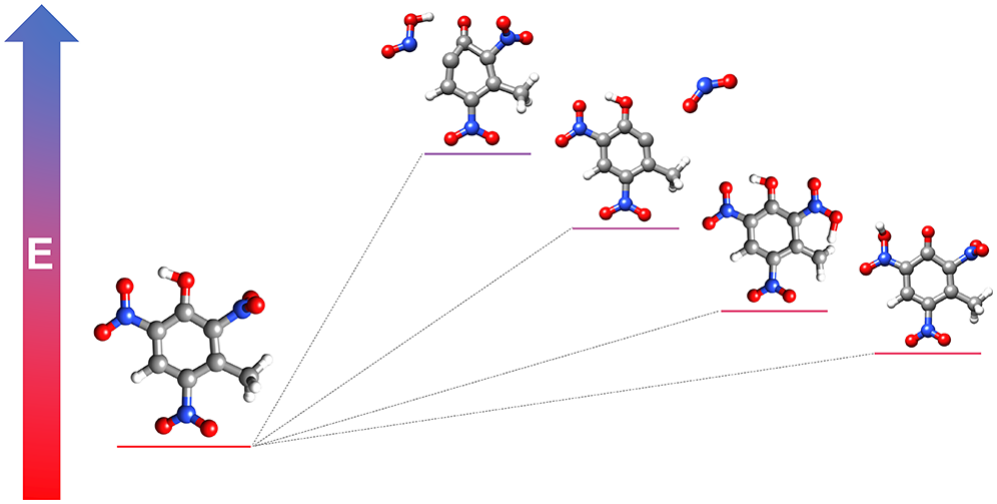

To handle energetic materials safely, it is important to have knowledge about their sensitivity. Density functional theory (DFT) has proven a valuable tool in the study of energetic materials, and in the current work, DFT is employed to study the thermal unimolecular decomposition of 2,4,6-trinitrophenol (picric acid, PA), 3-methyl-2,4,6-trinitrophenol (methyl picric acid, mPA), and 3,5-dimethyl-2,4,6-trinitrophenol (dimethyl picric acid, dmPA). These compounds have similar molecular structures, but according to the literature, mPA is far less sensitive to impact than the other two compounds. Three pathways believed important for the initiation reactions are investigated at 0 and 298.15 K. We compare the computed energetics of the reaction pathways with the objective of rationalizing the unexpected sensitivity behavior. Our results reveal a few if any significant differences in the energetics of the three molecules, and thus do not reflect the sensitivity deviations observed in experiments. These findings point toward the potential importance of crystal structure, crystal morphology, bimolecular reactions, or combinations thereof on the impact sensitivity of nitroaromatics.

## Introduction

Energetic materials are necessary ingredients in explosives, pyrotechnics, and propellants. Such materials must be handled safely when it comes to production, transportation, storage, use, and disposal. It is therefore of vital importance to know how sensitive they are to external stimuli such as impact or shock.^1^ Unfortunately, a desired high performance is inherently contradictory to low sensitivity,^2−4^ and the task of producing novel energetic compounds is furthermore complicated by the ever stricter environmental, health, and safety standards with which this class of materials must comply.

Although more than a century has passed since detonation phenomena were first observed,^5−8^ further development of consistent microscale theory of initiation is still of interest. While the literature surrounding energetic material sensitivity is abundant, fundamental understanding of the processes that govern sensitivity is still a matter of discussion.^9^ This is partly due to the very short time scales of energetic material decomposition, which makes it difficult to determine the specific reaction steps and to capture the underlying phenomena by standard experimental methods.^1,10^

Among the external stimuli challenging the stability of energetic materials is impact or shock.^1,11,12^ The impact sensitivity—one of the most common measures of sensitivity—is usually determined by the drop hammer test,^13−15^ where a hammer is dropped upon a sample of the material and the height at which an explosion is observed for some predetermined fixed percentage of the drops is recorded as the critical impact height. In the commonly employed Bruceton method,^16^ the critical impact height (*h*_50_) is defined as the distance from which 50% of the drops lead to explosion. While experimentally determined *h*_50_ values are heavily employed for correlation studies, it is well known that such results are associated with a high degree of uncertainty. This fact is to some extent due to the subjectivity of the human observer, which may severely influence the results—where one scientist may observe an explosion while another may not. Additionally, the measured values have proven to be quite sensitive to small changes in experimental setup and conditions.^1,17^ Thus, predictive theoretical models for impact sensitivity are desirable.

While the impact sensitivity depends on molecular properties related to the kinetics and thermodynamics of the decomposition reactions, solid-state properties such as particle size, polymorphism, crystal defects, and crystal orientation also play important roles.^13^ Defects are particularly important since they may form so-called hot spots under fast compression of the material. Although the initiation of an energetic material is a complex, multiscale process,^1,9,18,19^ one may roughly divide it into two main steps. The first step involves compression and deformation of the material, which leads to heating of the hot spots. In the second step, the material inside and surrounding the hot spots self-ignites and propagates into an explosion, provided that the hot-spot temperatures are sufficiently high.^13,20^ The temperatures in the hot spots can be significantly higher than the temperature in the bulk material. The ignition temperature of the hot spots is typically in the range of 300–1000 °C, and it is not the same as the thermal decomposition temperature.^21,22^

Over the last decades, advancements of computer technology and quantum chemical methods have provided an additional path to chemical insight. Consequently, computer simulations and molecular modeling have been widely^2,10,12,13,18,23−40^ employed in studies of energetic material sensitivity, and implementations of DFT are now among the most used tools in research on energetic materials. While these rapid technological advancements make it possible to study systems and processes that were previously inaccessible, important questions regarding the sensitivity of energetic materials remain unanswered.

In the quest for understanding, many^10,13,15,18,24−28,41−46^ have turned to correlation studies. In these studies, one or several descriptors (or functions thereof) are calculated or measured for a series of molecules and plotted against some function of the critical impact height. Among studied descriptors are electrostatic surface potential,^47−49^ nitro group charge,^28,50−52^ band gap,^43,46,53,54^ bond dissociation energy (BDE),^13,18,24−28^ heat of detonation,^13,44,55^ oxygen balance,^42,56^ and Wiberg bond indices.^10,32,57,58^ BDE is commonly used since it is easy to calculate and suitable to use in correlation calculations for impact sensitivity. Other factors, such as crystal packing, are harder to assess and consequently not as commonly described in the literature. While numerous correlations have been found, the obtained relationships are usually only valid for certain families of molecules, and outliers tend to occur.^10,13,42^ While correlation studies are helpful in determining standard behavior, they are less useful for the determination of mechanistic details. At least care must be taken in this respect, as the initiation of the decomposition of energetic materials may be complex.^19^ In the search for more detailed information about the phenomena that govern sensitivity, computational studies into plausible thermal unimolecular decomposition pathways of *ortho*-nitrotoluenes,^12,23,34,59^*ortho*-nitrophenols,^32,33,56^ and various other compounds have been performed. Additional computational and experimental studies have undertaken the possibility of bimolecular reactions in the early stages of decomposition in condensed-phase energetic materials.^39,60^

Nitroaromatic compounds comprise an important class of energetic materials^12,35^ and include several explosives of present and historical significance, such as 2,4,6-trinitrotoluene (TNT) and PA. The nitro group is an important explosophore whose presence as a substituent on the aromatic ring has been shown to sensitize energetic materials while at the same time enhancing their detonation performance.^14,18,23,60,61^ At temperatures above where the thermal stability of nitroaromatics is normally studied, three modes of initiation have been proposed: (a) homolytic cleavage of the weakest C–NO_2_ bond, (b) inter- or intramolecular hydrogen transfer to the nitro group, which in some cases may result in loss of HONO (nitrous acid) or water, and (c) NO_2_–ONO isomerization.^62^ The relative dominance of these decomposition pathways is believed to vary with temperature.^1^ Homolysis is a high-energy event, mainly observed at high temperatures.^1,23,60^ It represents a common assumption for the initiation of nitro-based explosives, called the trigger linkage hypothesis. This hypothesis states that the first step of the initiation is a bond cleavage and that the decomposition is triggered by the homolytic fission of an X–NO_2_ bond, where X is C, N, or O.^13,42^ Hydrogen transfer reactions may be arranged into different categories based on the origin of the transferred hydrogen atom.

Based on experimental and computational studies, intramolecular hydrogen transfer from the hydroxyl substituent to the neighboring nitro group has been proposed to be the dominant initiation step for *ortho*-nitrophenol and its derivatives at low to moderate temperatures.^1,32,33,56,63^ The C–H α-attack pathway is considered to play a similar role in the decomposition of *ortho*-nitrotoluene, TNT, and their derivatives.^12,23,36,60^ While the hydrogen transfer may result in the formation of HONO and a pentacycloketene derivative through what we in the following will refer to as the ketene-forming pathway, the α-attack gives anthranil (2,1-benzisoxazole) derivatives and water. For nitro derivatives of phenols and methylbenzenes, the above-mentioned NO_2_–ONO isomerization pathway has on several occasions^12,23,34^ been deemed to be of lesser importance than the C–NO_2_ homolysis and C–H α-attack pathways. Indeed, in a DFT study on TNT, Cohen et al.^23^ concluded that while NO_2_–ONO isomerization is thermodynamically favored over C–NO_2_ homolysis at room temperature, and more exergonic than both C–NO_2_ homolysis and C–H α-attack at high temperatures, it is kinetically unfavorable over the temperature range (298–3500 K) they studied. Consequently, the isomerization is only expected to contribute to TNT initiation in a minor fashion. Chen et al.^34^ drew the same conclusion, based on DFT calculations, that the NO_2_–ONO isomerization was less significant than other TNT decomposition pathways.

In studies on substituted nitroaromatics,^10,13,18^ it has commonly been assumed that the C–NO_2_ bond is the weakest one. Furthermore, experiments have revealed that C–NO_2_ homolysis occurs in the decomposition of *ortho*-nitrotoluene.^64−66^ Since impact sensitivity is a measure of the mechanical energy that must be provided to an energetic material to make it explode, it is considered closely connected to the activation energies of the initiation reactions. However, the C–NO_2_ homolysis pathway is usually studied by calculating the C–NO_2_ BDE, i.e., the reaction energy of the bond-breaking process, in contrast to other mechanisms for which the activation energy is the central quantity. There are several reasons why the BDE has become a common measure for studies on the sensitivity of energetic materials. First, the BDE has on many occasions been seen to correlate with impact sensitivity.^13,18,24,27^ Second, the activation energy of the homolytic bond-breaking process can be challenging to calculate since the corresponding transition state (TS) can be especially difficult to locate—or, in many cases, even nonexistent as the energy increases monotonically with the C–N distance until the dissociation is complete. This situation was found by Nikolaeva et al.^67^ who studied C–NO_2_ homolysis in nitrobenzene at the B3LYP/6-31G(d,p) level of theory. Third, it has been proposed that the BDE is proportional to—or even equal to—the activation energy for compounds where the resonance stabilization and the structure of the TS are relatively similar.^13^ Worth mentioning in this context are the results of Khrapkovskii et al.,^68^ who reported a significant correlation between the measured value of activation energy for C–NO_2_ homolysis in a variety of substituted nitroaromatics and the values of BDEs calculated at the B3LYP/6-31G(d,p) level of theory. The coefficient of determination, *R*^2^, was found to be 0.72. Several previous studies^12,23,34^ have compared the C–NO_2_ BDE with activation energies of other reaction mechanisms to identify the most favorable initiation process, and we choose to do likewise here.

While it is common to assume that C–NO_2_ homolysis initiates nitroaromatic decomposition, the experimental evidence for this mechanism is not as compelling for nitroaromatics as for other molecular families. Although NO_2_(g) has been observed upon decomposition of nitrobenzene at *T* = 548 K^69^ and from pyrolyzed 1,3-dinitrobenzene,^70^ 1,4-dinitrobenzene,^70^ and 1,3,5-trinitrobenzene^71^ by mass spectroscopy, NO_2_(g) is rarely observed from the decomposition of substituted nitrobenzenes in the bulk state.^1^ In an MS/MS-CID study by Yinon,^72^ early loss of NO_2_ was detected for TNT, but not for mPA. These observations suggest that substituted nitroaromatic compounds may have alternative decomposition channels with lower activation energies than those of C–NO_2_ homolysis. The presence of a hydroxyl substituent on a neighboring site to a nitro group opens the possibility of intramolecular hydrogen transfer from the former to the latter. Such a tautomerization, resulting in a quinoid or aci-structure, has been proposed as a possible reaction step in the early stages of decomposition of nitrophenols.

In a DFT study published in 2016, Vereecken et al.^40^ performed a comprehensive mapping of the ground and first excited state potential energy surfaces (PESs) of *ortho*-nitrophenol. Their results for the ground-state PES point toward the existence of several unimolecular mechanisms leading to the formation of cyclopentaketene and HONO. Ketenes are in general very reactive and are in this sense possible intermediates in a series of reactions leading to an explosion. One of the mechanisms reported in the study is a one-step process occurring via a single TS, whereas the remaining mechanisms involve multiple steps and include from one to three different aci-structures. For all of the multistep processes, the initial step is a tautomerization in which *ortho*-nitrophenol transforms to an aci-structure. This reaction step is associated with a relatively low activation energy for *ortho*-nitrophenol, which leads us to believe that it is an unlikely candidate for the rate-determining step (RDS) of the multistep decomposition of PA, mPA, and dmPA. Nevertheless, we have included this step in our work due to its frequent occurrence in the literature^32,36,56^ and also to compare the different pathways.

Similarly to how the ketene-forming pathway is considered important for the decomposition of *ortho*-nitrophenols, the C–H α-attack pathway is believed to play an important role in the decomposition of *ortho*-nitrotoluenes. There exist several clear indications that an α-CH bond ortho to the nitro substituent on an aromatic ring promotes the thermal decomposition processes of such compounds.^1,73−75^ The formation of anthranil has been observed for gas-phase *ortho*-nitrotoluene by laser-assisted homogeneous pyrolysis^64^ and in shock tube pyrolysis.^65,66^ Computational investigations on the feasibility of this pathway for TNT^23,34^ have indicated that it dominates the early phase of decomposition at temperatures below 1250–1500 K for this compound. To our knowledge, mPA and dmPA have not yet been subjects of such studies.

In 2007, Cohen et al.^23^ published DFT results showing that for TNT, the C–H α-attack pathway consists of four reaction steps, in which the first is a tautomerization reaction, the second is a rotation about a C–N bond, the third is a ring closure, and the fourth and final step is the elimination of water. While the activation energies associated with the initial tautomerization and the elimination of water were found to be similar in size, the ring closure reaction required less than half as much energy. The activation energy for the bond rotation (second reaction step) was not calculated but is assumed to be small relative to the others based on previous studies.^40,76,77^ Fayet et al.,^12^ when employing DFT for the study of substituted *ortho*-nitrotoluenes, found that internal hydrogen transfer might occur as an alternative to the bond rotation in the second step. Moreover, they found that the activation energy of this process was about half the magnitude of those associated with the tautomerization of the first step and H_2_O elimination of the last step. They also identified an additional reaction step in the mechanism. This step occurs after the initial tautomerization but prior to the bond rotation/hydrogen transfer and is a rotation about the N–OH bond whose activation energy was found to be insignificant (5 kJ mol^–1^).

Our work is focused on substituted trinitrophenols, specifically PA and its methylated derivatives mPA and dmPA. While PA is a historically important explosive that has been the subject of many previous studies, mPA and dmPA have not been investigated in such detail. In 1979, Kamlet and Adolph^42^ correlated critical impact height with oxygen balance and found the two latter compounds to be outliers. Despite having molecular structures that only differ from each other by one methyl substituent, mPA is astonishingly less sensitive than PA and even less sensitive than dmPA. The study of outliers may be a promising path toward increased understanding of underlying phenomena.^42^ The reported sensitivity variations between these structurally similar compounds aroused our curiosity and is the main concern of the present Article.

**Figure 1:**
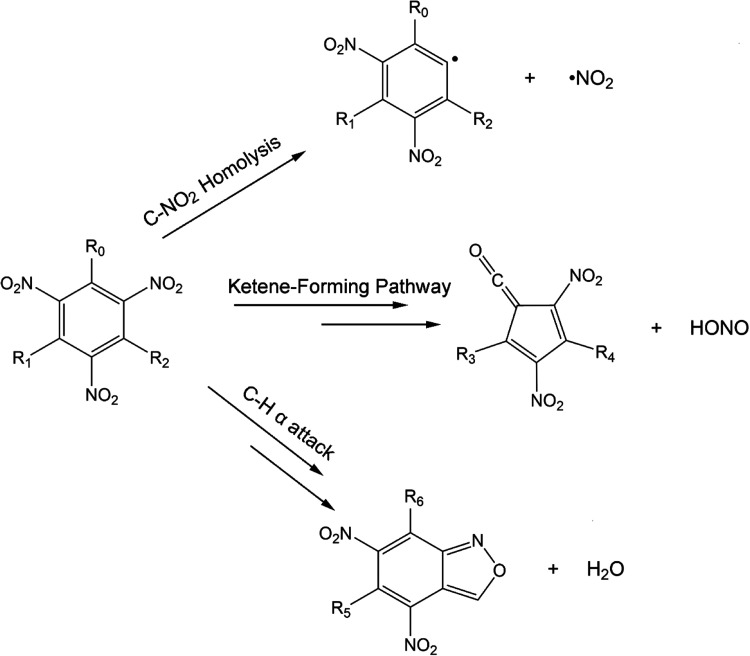
Schematic overview of the three reaction pathways studied for PA, mPA, and dmPA in the current work. The double arrows indicate that the reaction proceeds along a multistep pathway. The R groups (R_0_, R_1_, R_2_, R_3_, R_4_, R_5_, R_6_) are (OH, H, H, H, H, −, −) for PA, (OH, H, CH_3_, H, CH_3_, H, OH) for mPA, and (CH_3_, OH, CH_3_, CH_3_, CH_3_, OH, CH_3_) for dmPA.

The three decomposition pathways under study are sketched in Figure 1. The molecular structures of mPA and dmPA differ from that of TNT only by a hydroxyl substituent, and a hydroxyl and a methyl substituent, respectively. Moreover, it is assumed that neither the second nor the third reaction step identified by Cohen et al.^23^ or the additional step found by Fayet et al.^12^ is important for the overall rate of reaction for these molecules. For the current work, we therefore chose to investigate the initial tautomerization and the final H_2_O elimination steps only.

## Methods

All calculations are performed using the open-source computational chemistry software NWChem.^78^ Molecular graphics are constructed using UCSF Chimera,^79^ and energy plots are made using the MechaSVG software.^80^

DFT with the Minnesota hybrid functional M06-2X^81,82^ and the triple zeta valence polarized basis set def2-TZVP^83,84^ are employed throughout. The M06-2X functional has been shown to outperform B3LYP in energy calculations and in the computation of C–NO_2_ BDEs for a wide range of organic molecules.^85−88^ For geometry optimizations, M06-2X seems to yield results of similar accuracy to those obtained using B3LYP.^57,89,90^ With the additional knowledge that M06-2X is commonly employed in studies on energetic materials,^10,28,40,52,91^ applying it for the current work seems appropriate. To produce reliable vibrational frequencies, it proved necessary to employ a larger integration grid than the standard choice in NWChem.

Geometry optimizations are performed for all molecules treated in the current work (see Figures 3–5). Vibrational frequency calculations are employed to confirm each structure as either a minimum or saddle point on the PES, and to obtain the zero-point and thermal corrections to the molecular energies. The energy of each molecule at absolute zero temperature is obtained by adding the zero-point vibrational energy, scaled by a factor of 0.9754 in accordance with the results of Kesharwani et al.,^92^ to the electronic energy obtained in the final step of each geometry optimization. The Gibbs energies at temperature 298.15 K are obtained by adding to these electronic energies the enthalpy correction and subtracting the product of the computed entropy and the absolute temperature. No scaling factor is employed for the calculation of Gibbs energies.

The decision to calculate Gibbs energies at room temperature was made for the reason that at more realistic temperatures (>1000 K), the harmonic approximation breaks down. Also, most species exist in a spectrum of excited vibrational, and perhaps even electronic, states. A quantitative description of the effects of high temperatures from first principles is beyond the scope of the present contribution. The Gibbs energies presented nevertheless give a qualitative indication of the effect of temperature on the relative energy levels of the reaction paths for the molecules under study.

For each reaction step, the activation energy is calculated as the difference in energy (electronic energy + scaled zero-point vibrational energy for temperature *T* = 0 K, and Gibbs energy for *T* = 298.15 K) between the TS and the reactant while the reaction energy is found as the energy difference between the product and the reactant. The C–NO_2_ BDE, being the reaction energy of the homolytic bond-breaking process, is thus calculated as
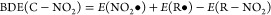
where the dot notation symbolizes radical species and R• is the radical that remains after the nitro group has been homolytically removed from the molecule.

## Results and Discussion

**Figure 2:**
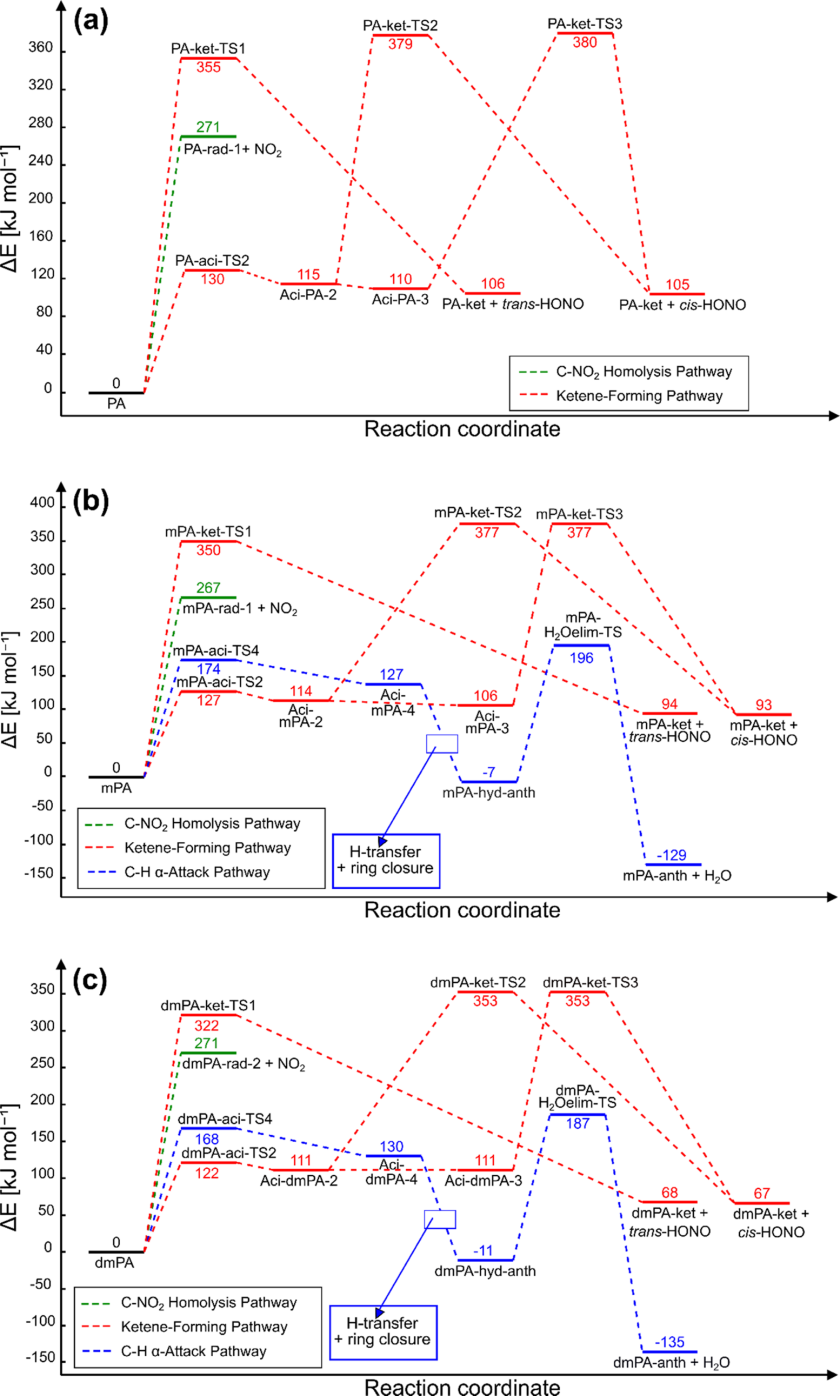
Energy plots for the investigated reaction pathways for (a) PA, (b) mPA, and (c) dmPA at temperature *T* = 0 K. See nomenclature in Figures 3–5. A barrier to the reaction Aci-Y-2 → Aci-Y-3 (Y ∈ {PA, mPA, dmPA}) is assumed but is not calculated since it is thought to contribute insignificantly to the total rate of reaction.

We consider the three pathways in detail by discussing them individually. For each of the studied compounds, an overview of the pathways is shown in the energy plots (*T* = 0 K) in Figure 2.

### C–NO_2_ Homolysis Pathway

**Figure 3:**
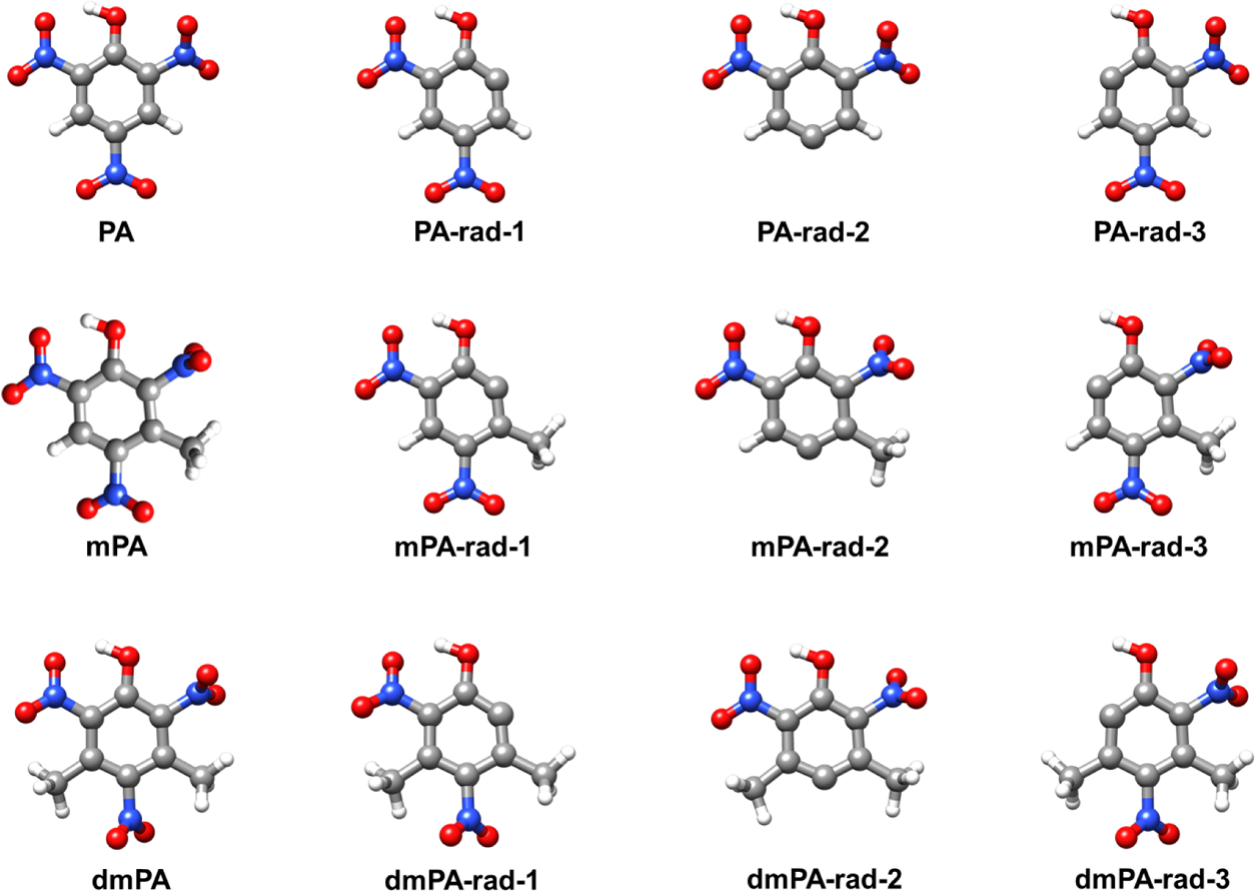
Optimized structures of the different stationary points (minima) investigated in the study of the C–NO_2_ homolysis pathway. The designations include the abbreviated name of the reacting molecule (PA, mPA, or dmPA), and “rad” is short for radical.

Figure 3 displays the optimized geometries and nomenclature of the structures treated in the study of the C–NO_2_ homolysis pathway.

For each compound, the C–NO_2_ bond that is found to have the lowest BDE is identified as the trigger bond in the gas phase. Table 1 displays the calculated trigger bond BDEs together with the compounds’ critical impact height (*h*_50_). The impact sensitivity data are from Kamlet and Adolph.^42^

**Table 1:** Calculated Trigger Bond BDEs (This Work) of PA, mPA, and dmPA, and Their Critical Impact Heights (*h*_50_), as Reported by Kamlet and Adolph^42^

molecule	BDE [kJ mol^–1^]	*h*_50_ [cm]
PA	271	87
mPA	267	191
dmPA	271	77

As seen in Table 1, the trigger bond BDEs are all found in the range of 267–271 kJ mol^–1^. This is in good agreement with the results of Shoaf et al.,^10^ who found the trigger bond BDEs of PA and mPA to be 270.0 and 263.6 kJ mol^–1^, respectively, at the M06-2X/TZVP level of theory. The lowest and highest BDE differ by only 4 kJ mol^–1^, and the trigger bond BDEs of PA and dmPA are equal. Based on various benchmark studies,^81,86^ 4 kJ mol^–1^ is believed to lie within the uncertainty area of the method. In other words, no significant variation in the BDEs is observed. The absence of a correlation between trigger bond BDE and impact sensitivity may be indicative of several phenomena. First, it is possible that some, or all, of the molecules decompose through a different reaction scheme. In contrast to the assumption that nitroaromatics decompose via C–NO_2_ homolysis at high temperatures and by other mechanisms at low to moderate temperatures, the anomalous impact sensitivities of mPA and dmPA may suggest that this is not the case for these two molecules. Second, the effects of solid-state properties like crystal structure and the density and nature of defects may to a great extent influence the impact sensitivity as discussed below.

### Ketene-Forming Pathway

With one exception, we examined all steps found to be associated with high activation energies for PA, mPA, and dmPA, as Vereecken et al.^40^ did for *ortho*-nitrophenol. In these steps, cyclopentaketene and HONO are formed from either the nitroaromatic molecule or an aci-structure. However, using the notation of Vereecken et al.,^40^ we did not investigate the step corresponding to the transformation of aci-NP-4 to cycloketene and HONO. The reason for this choice was that Vereecken et al.^40^ reported the activation energy to be significantly (about 30 kJ mol^–1^) lower than for the steps in which aci-NP-2 and aci-NP-3 transform to cycloketene and HONO. Thus, the mechanism including three different aci-structures is not treated in our work, nor is the step in which one aci-structure transforms into the other considered since its activation energy is assumed insignificant with regard to the overall reaction rate.

**Figure 4:**
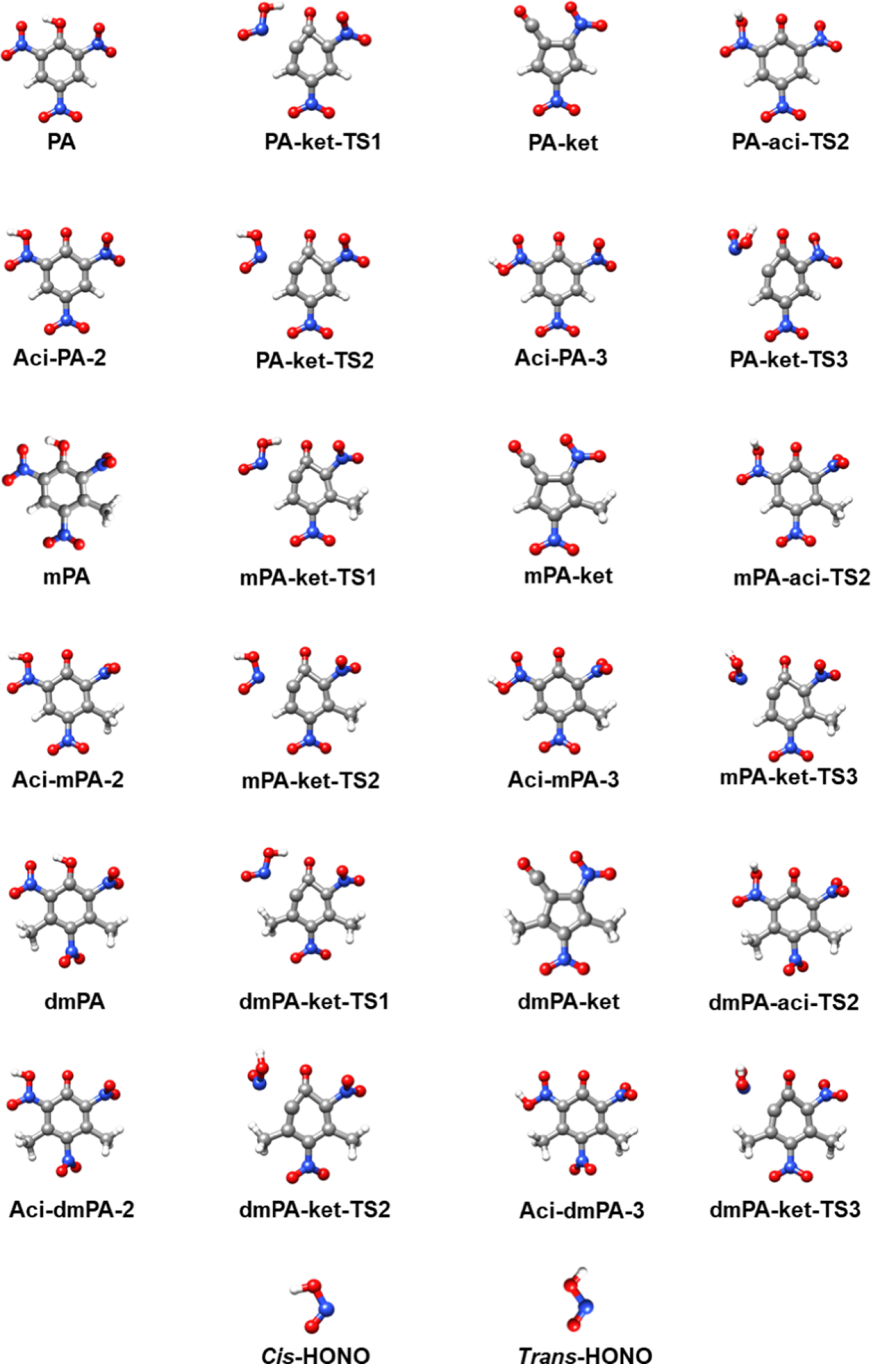
Optimized structures of the different stationary points (minima and TSs) investigated in the study of the ketene-forming pathway. The designations include the abbreviated name of the reacting molecule (PA, mPA, or dmPA), and “ket” is short for ketene.

Figure 4 displays the optimized geometries and nomenclature of the structures investigated in the study of the ketene-forming pathway. Inspection of each imaginary mode confirmed each TS to connect the desired reactants and products.

Energy plots for the ketene-forming pathway for PA, mPA, and dmPA are shown in Figure 2a–c, respectively. Both the one- and multistep processes found on the PES of *ortho*-nitrophenol by Vereecken et al.^40^ are identified for all three molecules. For each molecule, the largest activation energy is found for the one-step process in which HONO and Y-ket (Y ∈ {PA, mPA, dmPA}) are formed via a single TS. The barriers to the steps in which an aci-tautomer reacts to form HONO and Y-ket are, for Y ∈ {PA, mPA}, found to be about 90 kJ mol^–1^ lower than those associated with the one-step process. For Y = dmPA, the difference is about 80 kJ mol^–1^.

Under the assumptions that unimolecular processes are determining impact sensitivity and that PA, mPA, and dmPA all decompose through the same mechanism, one would expect the most sensitive compound (dmPA) to be associated with the lowest activation energies and the least sensitive compound (mPA) with the highest. The energy plots shown in Figure 2a,b reveal that for corresponding reaction steps of PA and mPA, the associated activation energies are essentially equal. For dmPA, however, the activation energies are found to be somewhat lower, as may be observed in the energy plot shown in Figure 2c. That is, for the tautomerization in which dmPA reacts to aci-dmPA-2, the differences are too small (8 kJ mol^–1^ with respect to PA and 5 kJ mol^–1^ with respect to mPA) to be significant. A larger difference in activation energies is found for the reaction steps in which the aci-Z compounds (aci-PA-Z, aci-mPA-Z, and aci-dmPA-Z), with Z ∈ {2, 3}, reacts to form HONO and cyclopentaketene derivatives (PA-ket, mPA-ket, and dmPA-ket, respectively). Here, the calculated barriers to reaction are found to be about 30 kJ mol^–1^ lower for dmPA than for PA and mPA.

Clearly, if one considers the high-barrier steps of the energy plots in Figure 2a–c simultaneously, the relative activation energies between molecules do not reflect the large variation in the species’ critical impact heights. The fact that PA and mPA are found to be associated with virtually equal activation energies while displaying significantly different sensitivity behavior may indicate that at least one of the molecules decomposes via another mechanism. For instance, it is possible that mPA and dmPA decompose via C–H α-attack—a mechanism unavailable for PA due to its lack of methyl substituents.

While the results raise questions about whether these molecules decompose via the ketene-forming pathway, it is still interesting to take a closer look at the tautomerization reaction that initiates the multistep versions of this mechanism. This reaction step has been studied for a variety of compounds containing adjacent nitro and hydroxyl groups, and correlations between impact sensitivity and activation energy have been reported.^32,56^ Due to the possibility that the first step is followed by bimolecular reactions in condensed-phase explosives, studying the first step may be fruitful. For this step, the activation energy obtained for each molecule is 130 kJ mol^–1^ for PA, 127 kJ mol^–1^ for mPA, and 122 kJ mol^–1^ for dmPA. A comparison of these values with the BDEs in Table 1 shows that the tautomerization requires only about half the energy that is needed to homolytically cleave the C–NO_2_ trigger bonds. This coincides with previous findings described by Oxley^62^ among others. However, the small variations between the molecules’ activation energies do not reflect the experimentally measured sensitivity differences.

Several studies have highlighted the first tautomerization step as central for the decomposition of energetic materials with neighboring nitro and hydroxyl substituents. By reaction force analyses, Murray et al.^33^ found the aci-tautomerization of PA to be feasible. However, they concluded that the PA ⇌ aci-PA equilibrium that is reached favors the nitro form. If the corresponding equilibriums of mPA and dmPA were to behave differently, this could hint toward an explanation for the differences in impact sensitivity. As may be observed in the energy plots in Figure 2, the barriers to the reverse reaction of the initial tautomerization are very low (≤15 kJ mol^–1^) in all cases. The reverse reactions are clearly kinetically favored over the reaction steps in which HONO and ketene derivatives are formed. The barriers have also been calculated for temperature *T* = 298.15 K (see below for a more detailed discussion), and they are all quite low. These results imply that PA, mPA, and dmPA all qualify for a reversible intramolecular hydrogen transfer. In other words, careful analysis of the first tautomerization step yields no explanation for the observed sensitivity differences.

### C–H α-Attack Pathway

**Figure 5:**
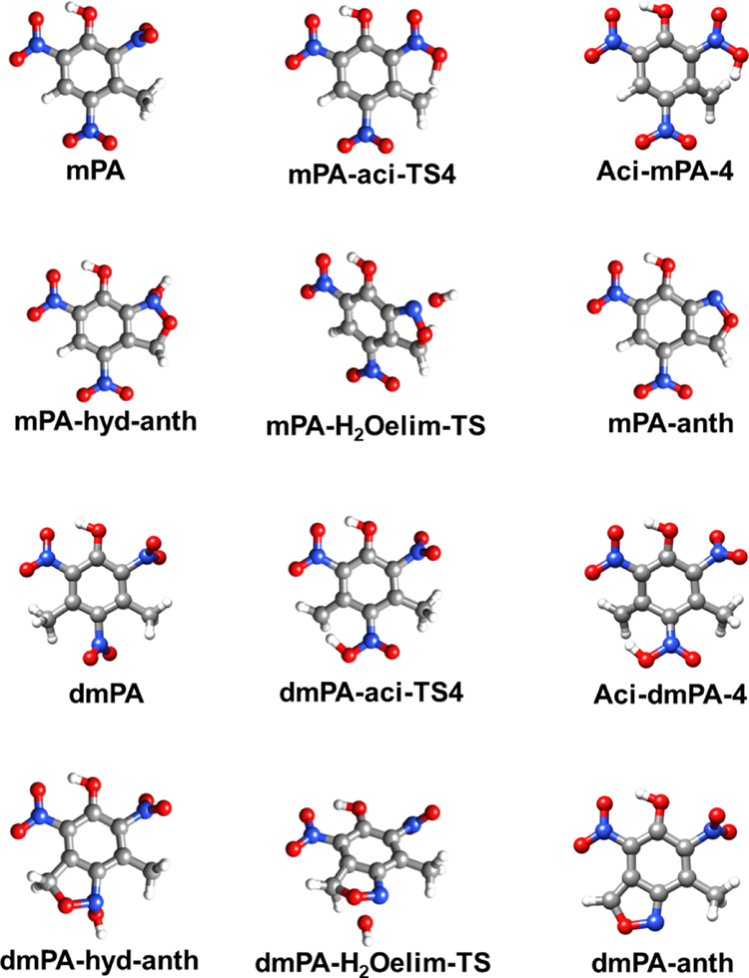
Optimized structures of the different stationary points investigated in the study of the C–H α-attack pathway. The designations include the abbreviated name of the reacting molecule (PA, mPA, or dmPA), and “H_2_Oelim” is short for water elimination.

Figure 5 shows the optimized geometries and nomenclature of the structures treated in the study of the C–H α-attack pathway. Inspection of each imaginary mode confirmed that each TS connects the desired reactant and product states.

In contrast to a hydroxyl substituent with two neighboring nitro groups, which due to its directionality may only donate its hydrogen in one direction, a methyl substituent with two neighboring nitro groups can donate a hydrogen atom in any of the two directions. Consequently, the C–H α-attack pathway may occur in two distinct ways in mPA, and four distinct ways in dmPA.

For mPA, the present results show that none of the two distinct ways in which the C–H α-attack pathway may occur is kinetically favored over the other. One of them is however slightly more thermodynamically favorable and is therefore chosen for further discussions. The species involved in this process are displayed in Figure 5. For the four distinct processes that may occur in dmPA, the lowest and highest activation energies found for the initial tautomerization step vary with 12 kJ mol^–1^ while those found for the H_2_O elimination vary with 9 kJ mol^–1^. Conveniently, the most kinetically favorable process is also thermodynamically favored. It is therefore selected for further discussions.

Figure 2b,c shows energy plots for the reaction steps of interest in the C–H α-attack pathway for mPA and dmPA, respectively. The blue-framed text boxes have been included to illustrate the intermediate steps (hydrogen transfer and ring closure reactions) that are not considered in the current work. In other words, aci-mPA-4 does not directly connect to mPA-hyd-anth, and neither does aci-dmPA-4 to dmPA-hyd-anth. In line with the findings of Cohen et al.^23^ for TNT at the uB3LYP/cc-pVDZ level of theory, the H_2_O elimination step is found to have the largest activation energy of the investigated reaction steps, for both mPA and dmPA. This also coincides with the findings of Khrapkovskii et al.,^59^ who concluded based on DFT calculations that for trinitrotoluenes with an α-CH bond, the RDS of thermal decomposition is not the initial tautomerization but rather a later reaction step.

By comparing the energy plots for the C–H α-attack pathway in Figure 2b,c, one may note that the barrier to the tautomerization of the first step is found to be 6 kJ mol^–1^ higher in mPA than in dmPA. For the H_2_O elimination of the final step, the difference in barriers is found to be 5 kJ mol^–1^. Upon assuming that the H_2_O elimination step is the RDS in the decomposition of these materials, one would certainly expect mPA to possess the highest activation energy of the two, due to being drastically less impact sensitive than dmPA. A difference of only 6 kJ mol^–1^ is, however, far too small to explain the different sensitivity behavior of mPA and dmPA. In fact, based on the results of previous benchmark studies^81,86^ it might even lie within the uncertainty range of the computational method.

### Dominant Reaction Pathways and Temperature Dependence

In Table 2, the activation energies for the main reaction steps at absolute zero are listed together with the values at 298.15 K. The results at the latter temperature show that the calculated trigger bond BDEs are essentially equal for PA, mPA, and dmPA. For the C–H α-attack pathway, the activation energies found for mPA and dmPA still only differ by 6 kJ mol^–1^ or less. Thus, as was found for absolute zero temperature, the results for these two pathways still do not reflect the large sensitivity differences observed in experiments. Finally, for the ketene-forming pathway, the activation energies of both the one-step process and the initial tautomerization step of the multistep processes show a trend that is somewhat consistent with the trend in the species’ impact sensitivities. Based on this, it may be tempting to assume that the ketene-forming pathway dominates the early stage of decomposition of these compounds and causes the anomalous sensitivity behavior. However, the following discussion will show that by assessing the energy changes of each pathway with the increase in temperature from 0 to 298.15 K, it seems unlikely that the ketene-forming pathway is kinetically favored at high temperatures.

**Table 2:** Activation Energies (in kJ mol^–1^) for the Main Reaction Steps at 0 K (Δ*E*_0_) and 298.15 K (Δ*E*_298_)^a^

		Y = PA		Y = mPA		Y = dmPA	
pathway	reaction step	Δ*E*_0_	Δ*E*_298_	Δ*E*_0_	Δ*E*_298_	Δ*E*_0_	Δ*E*_298_
C–NO_2_ homolysis	Y → Y-rad1 + NO_2_	271	219	267	219	271	218
ketene-forming	Y → Y-ket-TS1	355	340	350	368	322	306
	Y → Y-aci-TS2	130	123	127	152	122	111
	Aci-Y-2 → Y-ket-TS2	264	244	263	244	242	224
	Aci-Y-3 → Y-ket-TS3	270	262	271	259	242	235
C–H α-attack	Y → Y-aci-TS4			174	207	168	162
	Y-hyd-anth → Y-H_2_Oelim-TS			203	203	198	201

a Y denotes PA, mPA, or dmPA.

At both studied temperatures, the tautomerization step initiating the multistep versions of the ketene-forming pathway has the lowest activation energy of all reaction steps, for all three molecules. Thus, if one only considers the first step of each mechanism, that of the (multistep) ketene-forming pathway is kinetically favored over those of the other pathways at both temperatures. However, since both the ketene-forming and C–H α-attack pathways are multistep processes, and the ketene-forming pathway additionally has parallel reactions, it is more complicated to determine which pathway dominates for each molecule. On the one hand, the high-barrier steps of the ketene-forming pathway are found to have higher activation energies than does the assumed RDS of the C–H α-attack pathway. On the other hand, the products of the former pathway can be formed through all from one to four reaction steps, through four parallel reaction branches. The latter pathway may occur in two distinct ways in mPA and four distinct ways in dmPA; however, all of these distinct processes are composed of the same number of steps which are all crucial for the formation of products. Since impact initiation has been claimed to result in competitive contributions of different reaction pathways for several polynitroaromatics,^1^ caution is called for when drawing conclusions based on static gas-phase calculations.

For all three molecules, the most eye-catching difference is the lowering of the trigger bond BDE. This is unsurprising, as the formation of an additional molecule contributes six additional, easily excited, entropic degrees of freedom (rotational and translational). The reduction is 52 kJ mol^–1^ for PA, whereas it is 48 and 53 kJ mol^–1^ for mPA and dmPA, respectively. The activation Gibbs energies of the steps in the ketene-forming pathway for PA are lowered by 7–20 kJ mol^–1^. A similar reduction is calculated for dmPA. For mPA, the energies of the steps in which aci-mPA-2 and aci-mPA-3 transform to mPA-ket and *c*is**-HONO are also lowered (by 19 and 12 kJ mol^–1^, respectively). However, the activation Gibbs energies of the one-step process and the tautomerization of mPA into aci-mPA-2 are found to increase slightly when the temperature is increased.

Table 2 also reveals that the H_2_O elimination step in the C–H α-attack pathway has the highest activation Gibbs energy of this reaction sequence at both studied temperatures. In fact, the Gibbs energies of mPA-hyd-anth and mPA-H_2_Oelim-TS increase by an identical amount such that the activation energy is the same for both temperatures. However, the first step of this pathway is seen to increase with 33 kJ mol^–1^ with the change in temperature, which might indicate that this step becomes rate-determining for the C–H α-attack pathway at higher temperatures. According to the results of Cohen et al.,^23^ the activation Gibbs energy of the assumed RDS of the C–H α-attack pathway (i.e., the H_2_O elimination step) of TNT depends only slightly on temperature, while the C–NO_2_ homolysis pathway becomes increasingly exergonic as the temperature rises and the entropic contribution becomes more important. At the same time, they found the reaction barriers to the non-rate-determining steps of the C–H α-attack pathway to increase significantly with temperature, making C–NO_2_ homolysis kinetically favored. Thus, the temperature dependency of the C–H α-attack pathway seems to be similar for mPA and TNT. We found only small activation energy differences due to temperature increase for the steps in the C–H α-attack pathway for dmPA.

To summarize, the trigger bond Gibbs BDE is lowered to a significantly larger extent than the activation energies of the other reaction pathways, for all molecules. Additionally, this pathway consists of only one step, in contrast to the other mechanisms (except the one-step process of the ketene-forming pathway) for which multiple steps must occur for the products to be formed. These results may indicate that the C–NO_2_ homolysis pathway becomes kinetically favored for all molecules as the temperature is elevated further. Such a trend would coincide with chemical intuition, as well as the findings of several previous computational studies on TNT^23,34^ and *ortho*-nitrotoluene,^93^ which suggest C–NO_2_ homolysis to dominate the decomposition of these molecules for temperatures above ca. 1100–1500 K.

Based on experimental studies, Brill and James^1^ concluded that the initial decomposition step of PA involves the tautomer aci-PA-2 in the temperature range 418.15–623.15 K. Thus, there are indications that the ketene-forming pathway, or at least a part of it, is important for PA decomposition. However, the fact that the reaction is important in some temperature interval does not necessarily mean that it is relevant for impact sensitivity. That is, if an event that only occurs to a notable extent at temperatures that are too low to initiate the self-sustained exothermic reactions that eventually lead to an explosion, it cannot determine the impact sensitivity. However, it may be important for the general thermal stability of the material, which dictates how and for how long the material may be stored. That said, the importance of the ketene-forming pathway for the impact initiation of PA cannot be completely ruled out based on the present results.

An additional interesting finding is that the temperature dependency of the ketene-forming and C–H α-attack pathways seem to be different for mPA compared to the other molecules. The fact that the Gibbs energy of both the one-step process and aci-tautomerization step of the ketene-forming pathway are found to increase with temperature suggests that this pathway becomes less kinetically favored as the temperature rises. For the C–H α-attack pathway, the tautomerization in which mPA transforms to aci-mPA-4 is found to have an activation Gibbs energy only 12 kJ mol^–1^ lower than the trigger bond Gibbs BDE at temperature *T* = 298.15 K. Thus, by increasing the temperature from 0 to only 298.15 K, the difference in activation energy of these processes has decreased from 93 to 12 kJ mol^–1^. Based on this tendency, one can only assume that a further increase in temperature up to around 1000 K will both make the tautomerization mPA → aci-mPA-4 rate-determining for the C–H α-attack pathway and ensure that this pathway becomes the least kinetically favorable of them all. In total, these results suggest that the C–NO_2_ homolysis pathway becomes more strongly kinetically favored at a lower temperature than it does for PA and dmPA, and that the competition between the different mechanisms at elevated temperatures is stronger for the latter two compounds.

An important question that now needs to be addressed is whether the seemingly different temperature dependency of the ketene-forming and C–H α-attack pathways of mPA compared to that of the corresponding pathways of the other molecules can actually be linked to mPAs anomalously low impact sensitivity. This is the only qualitative difference found between mPA and the two other species from the present results. If the C–H α-attack pathway is crucial, this could possibly contribute to an explanation.

The crystal structures of mPA and dmPA are, to our knowledge, unknown. Properties related to the crystal structure of solid explosives have been shown to affect C–NO_2_ trigger bond BDEs as well as molecular charge distributions,^94^ two quantities that are routinely employed for correlation studies on impact sensitivity. The orientation of a molecule within the crystal with respect to its neighboring molecules affects the feasibility of intermolecular hydrogen transfer, while twisting of a single molecule’s functional groups affects the feasibility of intramolecular hydrogen transfer. Additionally, Trotter^95^ found that nitro group twisting occurring because of steric repulsions between functional groups lessens the resonance stabilization in some nitroaromatic molecules. Furman et al.^39^ showed how bimolecular reactions can lower the activation energy for decomposition of condensed-phase TNT and thus play an important role in the decomposition of this compound. Joshi et al.,^96^ employing solid-state MM/MD simulations for 1,3,5-trinitro-1,3,5-triazocyclohexane, focused on how energy transfer between phonon modes may govern the formation of hot spots. There is a possibility that similar studies of PA, mPA, and dmPA would yield similar results.

Based on our work and the literature, it is reasonable to assume that the variation in sensitivity between the three presently studied compounds cannot be explained by monomolecular phenomena. Intermolecular and/or solid-state effects are, hence, prime candidates for further study. One feature of the crystal structure important for impact sensitivity is crystal defects, which may form hot spots during the fast compression and deformation of the material, making it more sensitive. Thus, the structure and distribution of defects may influence sensitivity. Another feature of the crystal structure believed to correlate with sensitivity is face-to-face π-stacking. Ready sliding of this stacking might efficiently buffer against external stimuli and is therefore assumed to promote low impact sensitivity.^4,97−99^ Additionally, other solid-state properties such as particle size, crystal orientation, and polymorphism affect the sensitivity of an energetic material. Based on experimental results, Oxley et al.^60^ assume that the very low sensitivity of 1,3,5-triamino-2,4,6-trinitrobenzene (TATB) is due to solid-state effects. Specifically, they suggested that in the condensed phase, intermolecular attractive forces may be more important than the intrinsic molecular structure and that the thermal stability of TATB may be more a function of its lattice stability than an intrinsic property of the isolated molecule.^60^

## Conclusions

Upon assuming that PA, mPA, and dmPA decompose via the same reaction pathway, one would expect either the calculated trigger bond BDEs of the C–NO_2_ homolysis pathway or the calculated activation energies of the ketene-forming pathway to correlate with the impact sensitivities of the three compounds. We consider the computed energy differences to be too small to support such a conclusion. Considering how the energetics of the different pathways change with increasing temperature, it seems unlikely that the ketene-forming pathway dominates at elevated temperatures. Even if mPA and dmPA are assumed to decompose via the C–H α-attack pathway while PA follows one of the other reaction mechanisms, the results can still not explain the differences in sensitivities.

For all three compounds, the C–NO_2_ homolysis unsurprisingly seems to be the most temperature-dependent pathway. Combined with the fact that the C–NO_2_ homolysis is the only one-step pathway, it appears the most kinetically favored one at elevated temperatures.

There are several possible explanations why the results do not reflect the variation in sensitivities. First, the gas-phase calculations of the current work may not be representative of the studied pathways when the reactions occur in the condensed phases of the materials. Thus, it is possible that the pathways studied here are related to impact sensitivity even though no significant trends are revealed by the gas phase calculations. Second, the way the molecules are packed in the condensed phases, i.e., the crystal structure of each compound, may affect the decomposition processes in several ways. While twisting of functional groups out of the ring plane may affect both the C–NO_2_ bond strength and the feasibility of intramolecular hydrogen transfer, the orientation and geometry of each molecule with respect to its neighboring molecules affects the feasibility of bimolecular reactions. Furthermore, solid-state properties like particle size, crystal orientation, and polymorphism influence the sensitivity of an energetic material. Finally, the sensitivity may also be affected if the stacking of the molecules allows for sliding as a buffer against external stimuli.

The exact cause of the highly different impact sensitivities of PA, mPA, and dmPA has not been identified. However, the present results indicate that the cause of this unexpected sensitivity behavior is likely to be found in bimolecular reactions, crystal effects, or both.

## Supporting Information Available

Molecular geometries (ZIP)

## Notes

The .xyz-files for all molecular geometries can also be found at NTNU Open Research Data, DOI: 10.18710/NQKRKJ.
